# The effects of complex training on performance variables in basketball players: a systematic review and meta-analysis

**DOI:** 10.3389/fspor.2025.1669334

**Published:** 2025-09-24

**Authors:** Ibnu Noufal Kambitta Valappil, Koulla Parpa, Karuppasamy Govindasamy, Borko Katanic, Cain C. T. Clark, Masilamani Elayaraja, Debajit Karmakar, Alexandru Ioan Băltean, Patricia Roxana Forț, Vlad Adrian Geantă

**Affiliations:** ^1^ Department of Physical Education and Sports, Pondicherry University, Pondicherry, India; ^2^ Department of Physical Education, Govt. Higher Secondary School, Beypore, Kerala, India; ^3^ Faculty of Sport and Exercise Science, UCLan University of Cyprus, Pyla, Cyprus; ^4^ Department of Sports, Recreation and Wellness, Symbiosis International (Deemed University), Hyderabad Campus, Modallaguda (V), Nandigama (M), Rangareddy, Telangana, India; ^5^ Montenergin Sports Academy, Podgorica, Montenegro; ^6^ College of Life Sciences, Birmingham City University, Birmingham, United Kingdom; ^7^ Lakshmibai National Institute of Physical Education, Gwalior, Madhya Pradesh, India; ^8^ Faculty of Physical Education and Sport, Aurel Vlaicu University of Arad, Arad, Romania; ^9^ Doctoral School of Sport Science and Physical Education, Pitești University Center, National University of Science and Technology Politehnica Bucharest, Pitești, Romania

**Keywords:** change of direction speed, jump performance, plyometric, post-ActivationPotentiation, resistance training

## Abstract

**Introduction:**

Basketball requires explosive power, agility and change of direction (CoD) ability. Although often used interchangeably with agility, CoD is distinct: it involves rapid directional changes in response to a pre-planned stimulus, while agility also requires perceptual cognitive responses to unpredictable cues. In this review agility is considered under CoD, emphasizing the physical component that can be directly trained. Improving CoD and power is essential for optimal basketball performance. Complex training (CT), which combines strength and plyometric exercises, has emerged as a promising method. However, its specific effects on basketball player's physical performance variables remain unclear, warranting a focused systematic review and meta-analysis.

**Methods:**

A structured search strategy was conducted in accordance with the Preferred Reporting Items for Systematic Reviews and Meta-Analyses (PRISMA 2020) guidelines and the PICOS framework. PubMed, Web of Science, and Scopus databases were searched to identify appropriate Randomized Clinical Trials (RCTs) relating to CT in basketball players, up to May 2025. Standardised mean differences (SMDs), with 95% confidence intervals (CI), were calculated using a random-effects model. Heterogeneity (I^2^), sensitivity analysis, and publication bias were assessed using standard methods. Seven RCTs were included in the meta-analysis.

**Results:**

Analysing within-group effects following CT demonstrated significant improvements in CoD speed (SMDs: 1.11; 95%CI: 0.56 to 1.66; *p* < 0.001, *I^2^*:53), and vertical jump performance (SMDs: −1.44; 95%CI: −2.16 to −0.72; *p* < 0.001, *I^2^*:91). However, between-group comparisons (CT vs. active controls) revealed significant improvements only in CoD speed (SMDs: −1.04; 95%CI: −1.61 to −0.47; *p* < 0.001, *I^2^*:57) and vertical jump performance (SMDs: 1.01; 95%CI: 0.46 to 1.56; *p* < 0.001, *I^2^*:86). Funnel plot analysis indicated moderate asymmetry for CoD speed and clear asymmetry with outliers for vertical jump performance.

**Conclusion:**

Our findings demonstrate that CT significantly enhances jump performance and CoD speed in basketball players. This study highlights the efficacy of CT in significantly improving CoD speed and jump performance in basketball players. These findings support its inclusion in athletic conditioning programs and offer valuable insights for coaches and practitioners aiming to optimize sport-specific performance through targeted training interventions.

**Systematic Review Registration:**

https://www.crd.york.ac.uk/PROSPERO/view/CRD420251057718. PROSPERO (CRD420251057718).

## 1 Introduction

Basketball may be defined as a dynamic, intermittent team sport eliciting maximal effort through frequent accelerations, decelerations, sprinting, jumping, shuffling, and complex changes of direction (1, 2). Its substantial physiological demands involve high aerobic and anaerobic metabolic loads, with high-intensity actions relying on immediate energy sources (adenosine triphosphate [ATP], creatine phosphate [CP]) and sustained efforts utilizing glycolytic and aerobic pathways, respectively (1). Efficient energy system utilization and rapid recovery are crucial for maintaining peak performance, as fatigue impairs technical and tactical execution (3). Repeated high-intensity movements, including up to approximately 50 maximal vertical jumps per game, necessitate training that enhances power, speed, fatigue resistance, and recovery (3). Optimal basketball performance is inherently associated with high physical fitness, with muscular power often identified as a core determinant (4, 5). Indeed, common key performance variables include jump height and vertical jump, which are critical for rebounding, offensive plays, and shot contesting, reflecting explosive power, and were shown to be associated with performative success (5). Sprint speed was reported as pivotal for rapid acceleration, creating separation from defenders, facilitating fast breaks, and enabling quick defensive recovery (4). Whilst agility and change of direction (CoD) were described as complex athletic quality involving reaction speed, precision of movement, quick changes of direction, and rapid decision-making, all crucial for evading opponents and executing manoeuvres effectively (6). Finally, endurance was found to be critical for maintaining speed, strength, and focus throughout a competitive game, enabling repeated high-intensity actions, quick recovery, and sustained peak performance, which consequently reduces the likelihood of fatigue-related errors (3, 7).

Targeting the aforementioned core attributes offers synergistic benefits; for instance, strength and agility training were reported to contribute to injury prevention by building stronger musculoskeletal structures and improving postural control (8). Training approaches emphasize targeted plans to optimize performance variables, though the clamour for optimal, basketball-specific resistance training regimen to enhance diverse physical attributes continues (9).

Resistance training is widely recognized as fundamental for improving strength, hypertrophy, and power in basketball players (10). Research shows it enhances vertical jump, sprint speed, and stability, supporting rebounding, acceleration, and defense (10–12). Plyometric training, emphasizing the stretch–shortening cycle, develops explosive power, agility, and reactive strength, essential for rapid direction changes, fast breaks, and repeated jumps (13, 14). However, in isolation, each has limitations: resistance training mainly improves maximal force, while plyometric training emphasizes velocity and neuromuscular reactivity. This highlights the value of integrating both methods to maximize performance (15, 16). Complex training (CT), also referred to as contrast or post-activation potentiation training, was shown to improve explosive power by integrating high-load strength training (e.g., 75%–90% of one-repetition maximum, 1RM) with subsequent biomechanically similar plyometric exercises (17, 18). This contrast seeks to enhance power output in sport-specific tasks, like jumping and sprinting, by increasing fast-twitch muscle fibre recruitment (18).

For this review, CT specifically refers to this strength-plyometric pairing and its physiological basis. CT is prefaced on Post-Activation Potentiation (PAP), a transient increase in muscle force after intense contraction (17, 18). Putative mechanisms include phosphorylation of myosin regulatory light chains (MLC) and increased neural activation (19). While PAP is an acute phenomenon, its consistent application within a structured CT program was speculated to elicit more significant and lasting chronic adaptations in muscular strength and power (20). PAP effects were reported as highly individualized, with stronger and more highly trained athletes often demonstrating greater sensitivity (21). The fitness-fatigue model dictates optimal rest intervals (typically 3–12 min) between the heavy lift and the plyometric exercise to maximize potentiation over fatigue (22, 23). CT protocols typically alternate high-load weight training (75%–90% 1RM, 2–12 repetitions) with maximum intensity plyometrics (5–15 repetitions) within the same workout session. Rest intervals of 3–12 min between exercises are crucial for PAP (17). Beyond acute enhancement, repeated PAP exposure through structured CT programs is hypothesized to promote chronic adaptations such as improved neuromuscular efficiency, faster rate of force development, and enhanced recruitment of type II fibers qualities that directly underpin sprinting, jumping, and CoD ability in basketball (16). For chronic adaptations, effective CT programs were implemented over approximately 10 weeks, 2–3 sessions per week, with at least 48 h of recovery between sessions (17). The intensity of both components should remain high, but overall volume managed to prevent excessive fatigue and overtraining. The prevailing goal is chronic, long-term improvements, however variability in programming parameters indicates a need to identify optimal strategies.

The extant literature asserts CT improves jump performance (vertical, squat, countermovement jump), often more effectively than standalone training (24, 25). CT was reported to positively affect sprint abilities, both acutely and chronically. Whilst agility, CoD speed, muscular strength, isometric force, and explosive power, also responded positively, often surpassing traditional weight training (26, 27). Some studies also reported positive effects on aerobic and cardiorespiratory endurance (27). Furthermore, CT reportedly enhances vertical jump, sprint speed, agility, and muscular strength/power in basketball players and team sport athletes (16, 28). Despite positive findings, limitations and inconsistencies exist. Indeed, some studies suggest traditional training might be more effective in specific scenarios (18). Transferability of CT's general physical improvements to skill-specific aspects like shooting and passing is inconsistent; indeed, highly variable interindividual responses in youth players suggest individualized programs are crucial (29). There's a notable lack of research on open-skill agility or reactive movements, with most focusing on closed-skill agility, i.e., pre-planned (6, 30). Consensus on optimal CT protocols (rest intervals, intensity, volume) for basketball is lacking, as research often generalizes from other team sports (1). Concerns include excessive maximal strength training detracting from sport-specific conditioning or skill acquisition, whilst lower limb asymmetries in basketball players may also require specific attention (31). Although, several systematic reviews and meta-analyses have been conducted on basketball players using different training interventions (2, 30), while others have examined the effects of CT in various athletic populations (32, 33). Additionally, one systematic review has investigated CT in basketball players (1). However, no study to date has provided a comprehensive synthesis of the evidence on CT exclusively on performance variables in basketball players. Accordingly, given these persisting inconsistencies in CT research, the present study aimed to conduct a systematic review and meta-analysis to comprehensively evaluate the effects of CT on performance variables in basketball players.

## 2 Materials and methods

### 2.1 Review protocol

**Figure 1:**
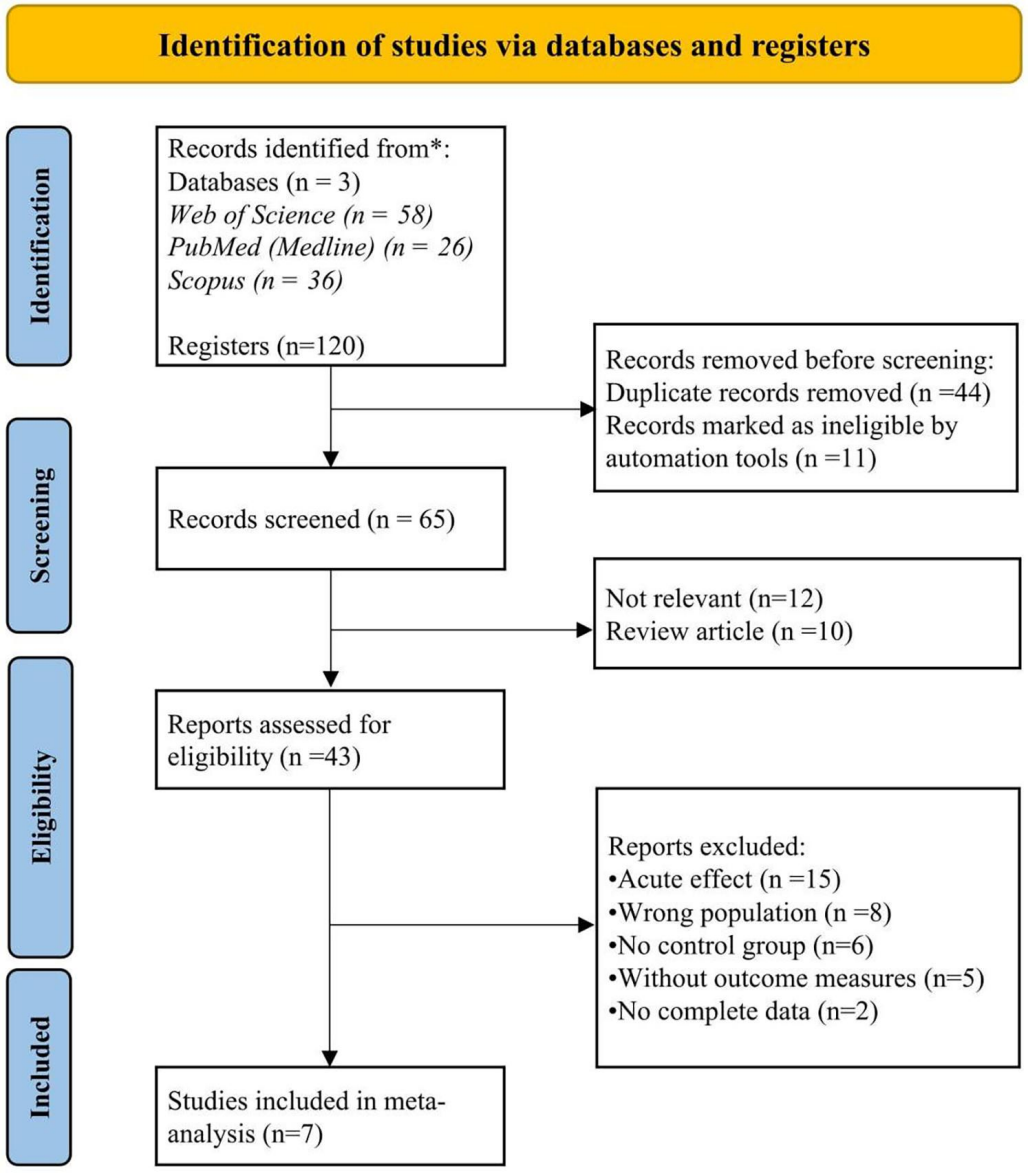
PRISMA flow diagram.

This systematic review and meta-analysis aimed to analyze the effects of CT on performance variables in basketball players. The review was carried out in line with the guidelines outlined in the Preferred Reporting Items for Systematic Reviews and Meta-Analyses (PRISMA 2020) (34) as illustrated in Figure 1. All procedures followed the recommended standards for transparency and methodological rigor. The review protocol was prospectively registered in the international prospective register of systematic reviews (PROSPERO; https://www.crd.york.ac.uk/PROSPERO/) to ensure research transparency and prevent selective reporting (Registration number: CRD420251057718).

### 2.2 Literature search strategy and inclusion, exclusion criteria

A computerized literature search was conducted on May 09, 2025, using a systematic approach based on the PICOS framework: Population, Intervention, Comparator, Outcomes, and Study design (35). Two independent reviewers (I.N.K.V and K.G) conducted the screening process. The search was carried out across three major electronic databases: MEDLINE (PubMed), Web of Science, and Scopus (36). Studies were included if they involved basketball players (Population); examined complex training, contrast training, French contrast training, or a combination of resistance and plyometric training within a single training session (Intervention); compared outcomes with an active control group (Comparator); and assessed at least one physical fitness parameter such as change of direction speed, agility, power (vertical jump performance outcomes). Only randomized controlled trials (RCTs) were considered (Study design) (37). Detailed selection criteria (Inclusion and exclusion based on PICOS) presented in Table 1 The search strategy included specific terms such as “complex training”, “contrast training”, “French contrast training”, “performance”, “strength”, “speed”, “jump”, “agility”, “change of direction”, and “basketball players”. Boolean logic was used to construct the search queries, incorporating a combination of keyword phrases, MeSH terms, and their logical combinations to ensure a comprehensive and targeted retrieval of relevant studies (Table 2) (38).

**Table 1:** Selection criteria used in the meta-analysis.

Category	Inclusion criteria	Exclusion criteria
Population	Basketball players	Non-basketball players, other healthy human and recreational active populations
Intervention	Complex training, contrast training, French contrast training, combination of resistance training and plyometric training in single training session	Other than complex training, contrast training, French contrast training, combination of resistance training and plyometric training in other than single training session
Comparator	Active control group	Other intervention, and passive control groups
Outcome	At least one measure of physical fitness including CoD speed, agility, power (vertical jump performance)	No physical fitness data, only injury prevention data
Study design	Randomized controlled trials (RCTs)	cross-sectional studies, controlled studies, case studies, and observational studies

**Table 2:** Detailed search strategies from selected databases.

Database	Search strategy	Number of The study
Scopus	TITLE-ABS-KEY (“complex training” OR “contrast training” OR “French contrast training” OR “ French contrast method” OR “combination training” OR “photoactivation potentiation” OR “PAP”) AND TITLE-ABS-KEY (“perfoarmance” OR “power” OR “strength” OR “speed” OR “sprint” OR “jump” OR “agility” OR “change off direction” OR “change-off-direction” OR “COD” OR “1arm” OR “physical fitness” OR “motor skills” OR “muscle power” OR “Jump” OR “explosive strength”) AND TITLE-ABS-KEY (“basketball” OR “basketball players”)	36
PubMed	(“complex training” OR “contrast training” OR “French contrast training” OR “ French contrast method” OR “combination training” OR “postactivation potentiation” OR “PAP”) AND (“performance” OR “power” OR “strength” OR “speed” OR “sprint” OR “jump” OR “agility” OR “change of direction” OR “change-of-direction” OR “COD” OR “1RM” OR “physical fitness” OR “motor skills” OR “muscle power” OR “Jump” OR “explosive strength”) AND (“basketball” OR “basketball players” [MeSH])	26
Web of Science	TS = ((“complex training” OR “contrast training” OR “French contrast training” OR “ French contrast method” OR “combination training” OR “postactivation potentiation” OR “PAP”) AND (“performance” OR “power” OR “strength” OR “speed” OR “sprint” OR “jump” OR “agility” OR “change of direction” OR “change-of-direction” OR “COD” OR “1RM” OR “physical fitness” OR “motor skills” OR “muscle power” OR “Jump” OR “explosive strength”) AND (“basketball” OR “basketball players”))	58

### 2.3 Eligibility criteria

The inclusion criteria for the present systematic review and meta-analysis were as follows: (1) studies involving basketball players as participants, regardless of gender, age category, or performance level; (2) application of CT, contrast training, French contrast training, or a combination of resistance and plyometric training within a single training session as the intervention (39); (3) use of an active control group for comparison, which may include standard training practices, traditional resistance training, plyometric training alone, or other exercise modalities designed for performance enhancement; (4) investigation of at least one measure of physical fitness, including change of CoD speed, agility, vertical jump performance, or; and (5) inclusion of RCTs published in peer-reviewed journals. Articles were included irrespective of publication year, provided full-text access was available. Studies were excluded if they: (1) involved non-basketball players, other healthy populations, or recreationally active individuals outside the sport of basketball; (2) employed interventions other than the specified training types or implemented the combined resistance and plyometric training in a format other than a single session; (3) used passive control groups with no training or minimal physical activity; (4) did not report any relevant physical fitness outcomes or focused solely on injury prevention; and (5) were not RCTs, such as cross-sectional, controlled non-randomized, observational studies, case reports, or studies lacking a clearly defined control group (Table 1). At least three studies were identified for each outcome measure included in the meta-analysis (40, 41). However, due to a lack of eligible articles on muscular strength and linear sprint performance, this outcome was excluded from the meta-analysis.

### 2.4 Selection process and data extraction

The study selection process for this meta-analysis on CT began with the identification of records through comprehensive searches in three electronic databases. An AI-powered automation tool (https://www.rayyan.ai/) was used to streamline the initial filtering by simultaneously removing duplicates and excluding clearly irrelevant and review articles based on titles and abstracts (42). The remaining full-text articles were then reviewed for eligibility using the predefined PICOS framework (Population, Intervention, Comparator, Outcome, and Study Design). Two reviewers (I.N.K.V and K.G) independently conducted the screening process, In cases of disagreement or uncertainty, a third reviewer (B.K) applied the same methodology independently to resolve any conflicts. Eligible studies were then included for data extraction, which was independently performed by two reviewers (I.N.K.V and K.G), collecting details such as the first author's name, year of publication, country, study design, sample size, intervention characteristics (type, duration, frequency. and follow-up), outcome measures, and main findings (Table 3). A third reviewer (B.K) verified the extracted data to ensure accuracy and completeness. The study selection procedure is visually presented in the PRISMA flow diagram (Figure 1).

**Table 3:** Study characteristics.

Study	Country	n	Age (years)	Players level	Duration (week)	Intervention	Control	F/W	Specific physical fitness outcomes
Biel et al. (44)	Poland	CT = 13 CMP = 11	17–30	Semi-professional male basketball players	8	Complex training	Compound training	2	Vertical jump performance (CMJ) ↑
Freitas et al. (46)	Spain	MCT = 9 OLT = 9	17–26	Semi-professional male basketball players	6	Modified complex training	Optimal load training	2	CoD (T-Test) ↓ Vertical jump performance (CMJ) ↑
Hassan et al. (49)	Saudi Arabia	CRCT = 12 CT = 12 CRT = 12	17–20	Amateur male basketball players	10	Core complex training and complex training	Core training	3	Vertical jump performance (SJ) ↑*
Latorre Román et al. (45)	Spain	CCT = 30 CG = 28	8–10	Academy basketball players (Pubertal Stage boys and girls)	10	Contrast training	Regular training	2	CoD (*T*-Test) ↓* Vertical jump performance (SJ, CMJ, DJ-20, and DJ-40) ↑*
Sánchez-Sixto et al. (15)	Spain	CT = 13 PLY = 11 CG = 12	15–30	Competitive female basketball players	6	Complex training	Plyometric training and regular training	2	Vertical jump performance (CMJ)↑*
Santos et al. (47)	Portugal	CT = 15 CG = 10	14–15	Young male basketball players	10	Complex training	Regular training	2	Vertical jump performance (SJ, CMJ, ABA, and DJ) ↑*
Wang et al. (48)	China	CT = 16 RT = 16	16–23	Female college basketball players	8	Complex training	Resistance training	2	CoD (IAT, and 505) ↓* Vertical jump performance (CMJ) ↑*

n, Number of subjects in each group; F/W, Frequency per week; CT, Complex training; CMP, Compound training; MCT, Modified complex training; OLT, Optimum load training; CRCT, Core complex training; CRT, Core training; CCT, Contrast training; CG, Control group; PLY, Plyometric training; RT, Resistance training; CMJ, Countermovement jump; CoD, Change of direction; DJ, Drop jump; SJ, Surgent jump; ABA, Abalakov test; IAT, Illinois agility test; ↑, Value increased; ↑*, Value significantly increased; ↓, Value decreased; ↓*, Value significantly decreased.

### 2.5 Assessment of methodological study quality

The methodological quality of the seven RCTs included in this meta-analysis was assessed using the Physiotherapy Evidence Database (PEDro) scale, which consists of 11 dichotomous (yes/no) items that evaluate key methodological criteria, including random allocation, allocation concealment, baseline comparability, blinding (of participants, therapists/researcher, and assessors), adequacy of follow-up, intention-to-treat analysis, between-group statistical comparisons, and reporting of point estimates with measures of variability (43). Two independent reviewers (I.N.K.V and K.G) conducted the assessments, resolving any disagreements through consultation with a third reviewer (B.K). Reviewers were not blinded to the study authorship, journal of publication, or study outcomes. The PEDro scores of the included studies were as follows: Biel et al. (44) and Latorre Román et al. (45) scored 6 (classified as good quality), Freitas et al. (46), Sánchez-Sixto et al. (15), Santos et al. (47), and Wang et al. (48) scored 5 (fair quality), and Hassan et al. (49) scored 4 (poor quality. Although scores of 5 and 6 indicate fair to good methodological quality according to PEDro guidelines, they do not reflect full methodological rigor and highlight areas of potential bias or insufficient reporting within the included studies (Table 4).

**Table 4:** Methodological quality score of the studies included in the meta-analysis (PEDro).

References	Items											Total
	1	2	3	4	5	6	7	8	9	10	11	
Biel et al. (44)	✓	✓	X	X	X	X	✓	✓	✓	✓	✓	6
Freitas et al. (46)	✓	✓	X	X	X	X	X	✓	✓	✓	✓	5
Hassan et al. (49)	✓	✓	X	X	X	X	X	X	✓	✓	✓	4
Latorre Román et al. (45)	✓	✓	X	✓	X	X	X	✓	✓	✓	✓	6
Sánchez-Sixto et al. (15)	✓	✓	X	X	X	X	X	✓	✓	✓	✓	5
Santos et al. (47)	✓	✓	X	✓	X	X	X	X	✓	✓	✓	5
Wang et al. (48)	✓	✓	X	X	X	X	X	✓	✓	✓	✓	5

Scores of 4 are considered “poor”, 4 to 5 are considered “fair”, 6 to 8 are considered “good” and 9 to 10 are considered “excellent”.

### 2.6 Data extraction

Data related to physical fitness variables-including CoD speed, and vertical jump performance were extracted from each included study. CoD speed was collected form the *T*-Test, Illinois Agility Test (IAT), and the 505 CoD test (45, 46, 48). Vertical jump performance was collected form such as the countermovement jump (CMJ), squat jump (SJ), drop jump (DJ), and Abalakov jump (ABA) (44–46, 47, 48). One author (I.N.K.V) extracted the means, standard deviations (SD), and sample sizes (n) from the included articles, and a second author (K.P) independently verified the accuracy of the extracted data. Any discrepancies between the two researchers were resolved through discussion with a third author (K.G).

## 3 Statistical analyses

All statistical analyses were conducted using a random-effects model in Review Manager software (RevMan, version 5.4.1), employing the non-Cochrane mode, which provides access to the Meta View module, which facilitates comprehensive data visualization for meta-analytical outcomes (50). Statistical significance for all outcome measures was set at *p* ≤ 0.05. A random-effects model using the inverse-variance method was applied, as this approach allocates weights to individual studies based on the precision of their effect estimates, determined by the magnitude of their standard errors, and accounts for potential heterogeneity across included studies (51). Effect sizes were reported as standardized mean differences (SMDs), accompanied by 95% confidence intervals (Cls). The SMD was calculated using the formula SMD = (M_1_ - M_2_)/SD _pooled_, where M_1_ – M_2_ denotes the mean difference between intervention and control groups, and SD _pooled_ represents the pooled standard deviation (52). The SMDs (magnitude of effect sizes) was interpreted according to the following thresholds: trivial (0–0.2), small (0.2–0.5), moderate (0.5–0.8), and large (>0.8) (53). In studies involving multiple intervention groups, the sample size of the control group was proportionally divided to ensure appropriate comparisons. Heterogeneity among studies was assessed using the I^2^ statistic, with values of <25%, 25%–75%, and >75% interpreted as indicating low, moderate, and high heterogeneity, respectively (54).

## 4 Results

### 4.1 Trial flow

The initial search conducted by the first author (I.N.K.V) yielded 120 records related to the predefined study keywords. Preliminary filtering, carried out by the second author (K.P) using an Rayyan automated tool (https://www.rayyan.ai/). removed 44 duplicate entries and 11 ineligible records. Following this step, 65 potentially relevant articles remained (see Figure 1). These records were subsequently screened by authors I.N.K.V and K.G in Rayyan automated tool for relevance based on their titles; in cases where relevance was unclear, abstracts were also reviewed to ensure accurate classification. Titles deemed unrelated to the topic were excluded, although abstracts were frequently examined as an added measure to ensure thoroughness. As a result, 12 records were excluded due to irrelevance, and 10 were excluded for being review articles. The remaining 43 articles underwent full-text screening by authors I.N.K.V, K.G, and C.C.T.C. Of these, 36 studies were excluded for not meeting the inclusion criteria based on PICOS framework or for being published in languages other than English. Ultimately, seven RCTs met the eligibility criteria and were included in the final meta-analysis (Figure 1).

### 4.2 Study population and quality

A total of 229 healthy and athletic participants (Basketball players) ranging in age from 8 to 30 years were involved in the seven trials included in this Meta-analysis. Of the total sample, 25.32% were adolescent boys and girls, 29.69% were female, and 44.99% were male participants. The participants were predominantly basketball players at various competitive levels, including semi-professional, amateur, academy-level (pubertal boys and girls), and college athletes, with representation from both male and female cohorts. The average sample size per study was approximately 32.71 participants, and least participants in single group was 9 and most was 30. All trials included two to three study arms comparing complex or contrast training interventions against various control conditions such as regular training, resistance training, plyometric training, core training, and optimal load training. The duration of the interventions ranged from 6 to 10 weeks, with training frequencies of two to three sessions per week (Table 3).

### 4.3 Risk of bias assessment

**Figure 2:**
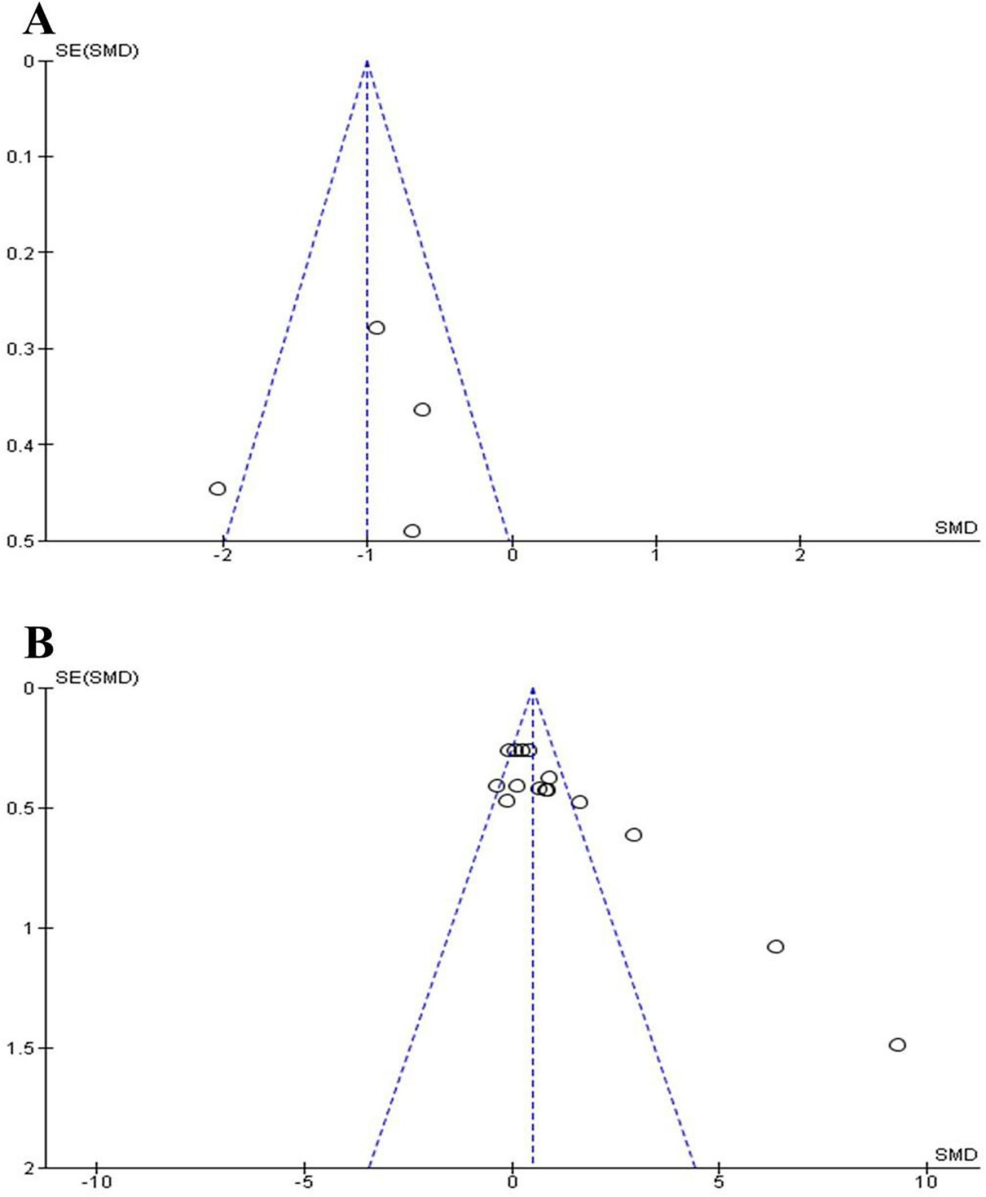
Funnel plots for publication bias assessment on (A) change of direction speed; (B) vertical jump performance.

Figure 2 present funnel plots assessing publication bias for four outcome measures: CoD speed (A), and vertical jump performance (B). The plot for CoD speed shows moderate asymmetry with a skew toward studies reporting improved performance, suggesting potential publication bias, though with limited data points (55). vertical jump performance displays clear asymmetry and an extreme outlier, indicating evident publication bias likely favouring studies with large positive effects (56).

### 4.4 Meta-analysis on change of direction speed

**Figure 3:**
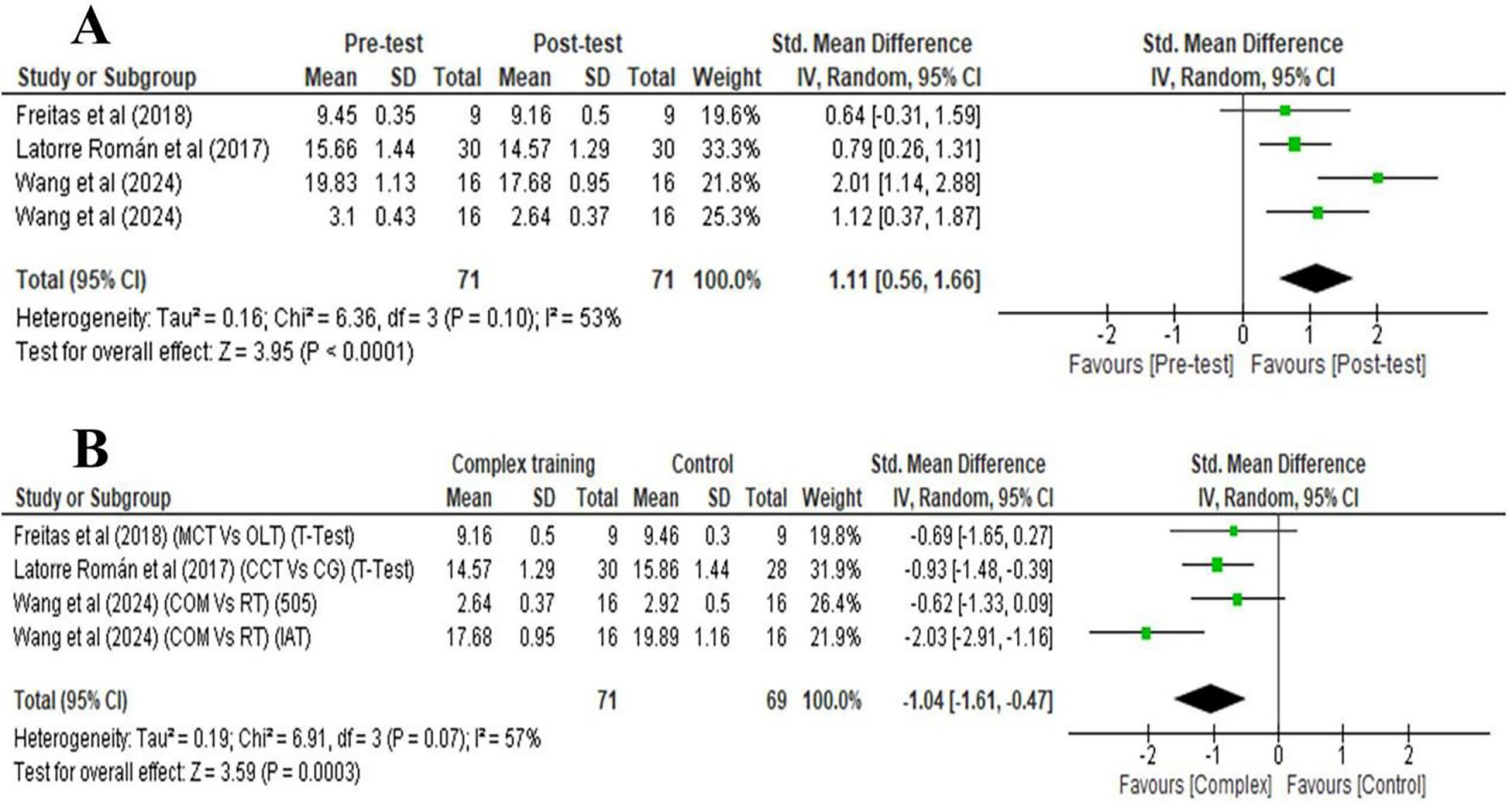
Forest plots depicting CoD outcomes: **(A)** Pre- and post-test differences following complex training; **(B)** post-intervention comparison between complex training and control groups.

A very large significant improvement was noted after CT [pre-post analysis (within group)] for CoD speed [two studies: SMDs = 1.11, 95% of Cl (0.56, 1.66), *p* < 0.001] (Figure 3A). Figure 3B reveals that there is a significant difference was observed between the CT group and the active control group for CoD speed, as indicated by Two studies with SMDs of −1.04 (very large effect), with a 95% confidence interval (CI) of −1.61, −0.47, and a *p*-value of 0.0003 (*p* < 0.001).

### 4.5 Meta-analysis on vertical jump performance

**Figure 4:**
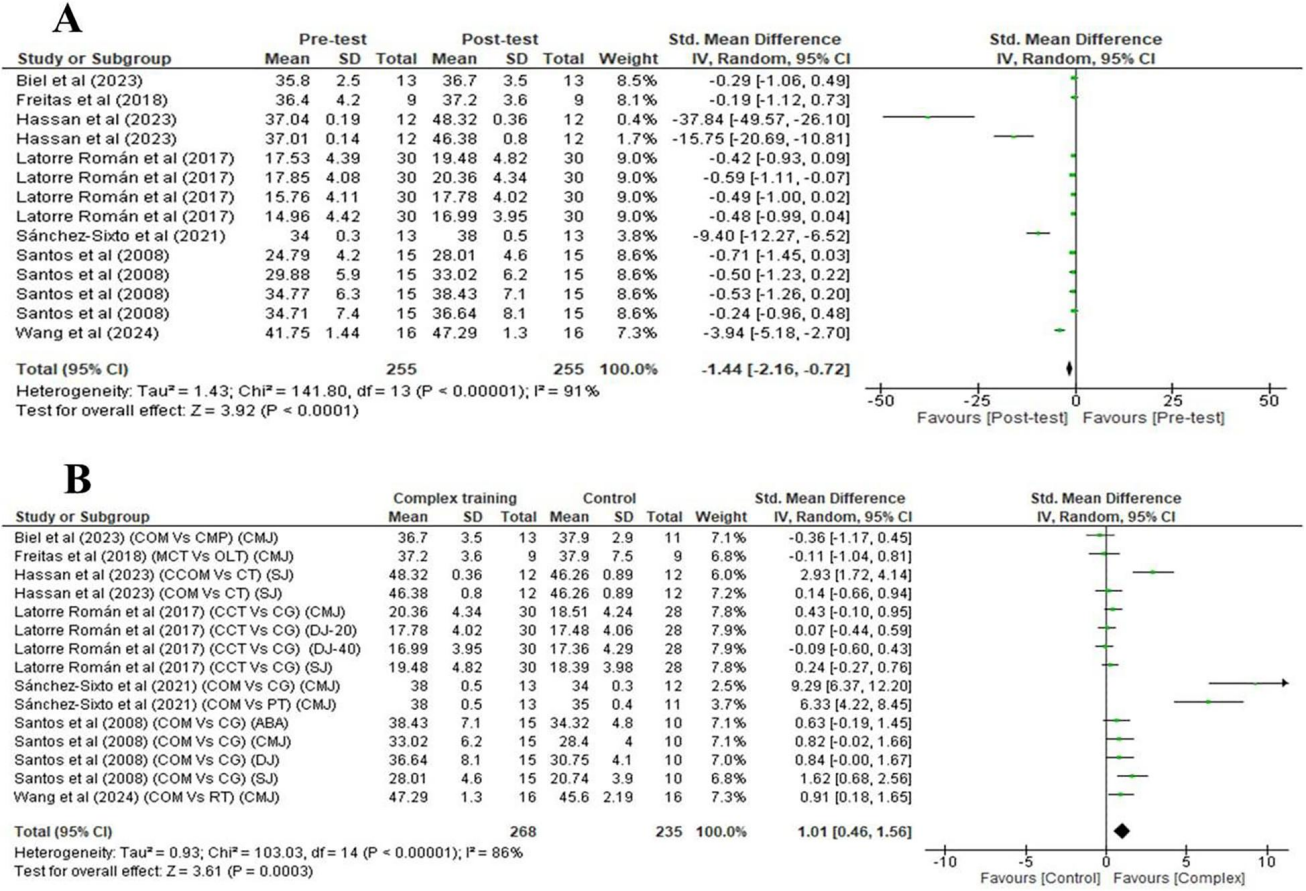
Forest plots depicting vertical jump performance: **(A)** Pre- and post-test differences following complex training; **(B)** post-intervention comparison between complex training and control groups.

A very large significant improvement was noted after CT [pre-post analysis (within group)] for vertical jump performance [three studies: SMDs = −1.44, 95% of Cl (−2.16, −0.72), *p* < 0.001] (Figure 4A). Figure 4B reveals that there is a significant difference was observed between the CT group and the active control group for vertical jump performance, as indicated by three studies with SMDs of 1.01 (very large effect), with a 95% confidence interval (CI) of 0.46, 1.56, and a *p*-value of 0.0003 (*p* < 0.001).

Figures 3, 4 illustrate that heterogeneity in physical performance measures following CT (within-group pre-post analysis) varied from moderate to high across different variables, with I^2^ values 53% for CoD speed, and 91% for vertical jump performance Similarly, heterogeneity between the CT group and the active control group also ranged from low to high, with I^2^ values of 57% for CoD speed, and 86% for vertical jump performance.

## 5 Discussion

This systematic review and meta-analysis aimed to evaluate the effects of CT on physical performance variables in basketball players. The findings revealed that CT had a significant positive impact on CoD speed and vertical jump performance. These results suggest that CT is effective in enhancing overall physical performance, although its advantages may vary depending on the specific performance domain when compared to other training methods. Several underlying physiological and neuromuscular mechanisms may contribute to these observed outcomes. The improvements observed in CoD performance following CT can also be attributed to neural adaptations and enhanced motor unit recruitment. These adaptations are essential for producing rapid, high-force muscle actions required during CoD tasks (57). Effective CoD ability involves rapid force development, strong eccentric control, and efficient transition from eccentric to concentric contractions in the lower limb musculature especially the quadriceps and hamstrings (11, 13, 58). Plyometric exercises, which are integral to CT, specifically target these mechanisms by improving leg stiffness, reactive strength, and neuromuscular coordination (39).

Importantly, CT enhances the neuromuscular coordination and intra- and inter-muscular synchronization necessary for executing basketball-specific movements, such as rebounding, defensive slides, and rapid directional changes (1). The increased recruitment and synchronization of type II muscle fibers promote faster contractions and greater force output, critical for both jumping and agile movements (59). Thus, the combination of strength and plyometric elements in CT supports not only muscular strength but also the neuromechanical efficiency needed for high-performance athletic movements (45). Some of the previous literatures are consistent with findings of the current study (60, 61). One of the meta-analyses conducted by Thapa et al. (60), explore that CT demonstrated superior improvements in CoD speed compared to traditional resistance training (RT). Similarly, Cormier et al. (61), reported that both complex and contrast training demonstrated superior effects on lower-body strength, vertical jump, sprint speed, CoD speed. These studies collectively suggest that the synergistic effect of combining resistance and plyometric exercises maximizes the transfer of strength gains into sport-specific, explosive, and multidirectional movements (60, 61).

A meta-analysis investigating the association between CoD speed and lower-extremity power concluded that there is a predominantly negative and moderate relationship between these variables. Specifically, higher lower-body power output is significantly associated with improved (i.e., faster) CoD performance, suggesting that enhancing lower-limb power can effectively contribute to greater agility in athletes (62). The study results indicate that CT enhances vertical jump performance, a benefit that can be partly explained by the PAP theory. Plyometric exercises applied after resistance exercise increase intramuscular activity and allow more motor units to be activated. This situation increases the activation of type II (fast twitch) muscle fibers and enables the muscle to produce higher power. As a result, the individual can produce more force during the jump (18). Consequently, basketball players can achieve higher vertical jumps and more explosive take-offs during plays such as jump shots or rebounds (63).

CT can improve the efficiency of the neuromuscular system by increasing motor unit synchronization and firing frequency (23). This situation may have positively affected the muscle contraction speed and force production time. The sequential combination of resistance and plyometric exercises facilitates rapid force application at basketball-specific movement velocities, promoting better performance in explosive actions such as jumping (16). CT is a training method that includes plyometric exercises. Therefore, the actions performed during plyometric exercises may have enabled force and movement transfer. While resistance exercises increase muscle strength, subsequent plyometric exercises (e.g., depth jumps) teach the rapid application of force to the ground. The sequential use of these two training modalities may have facilitated the use of maximal force at the specific speed of movement and increased its transfer to jumping performance (1). Furthermore, CT may have decreased the amortization time (ground contact time) by increasing the stiffness of the muscle-tendon complex (64). Since shorter contact time increases reactive force production, CT may have contributed to the athletes' jump height. Many studies in the literature support these results (16, 32, 65). Studies reveal that combined training approaches play a fundamental role in improving jumping performance. For example, a systematic review study conducted by Uysal et al. (16) emphasizes that combined training, including strength training, improves vertical jump height more than plyometric training alone, especially in basketball players over 18. One of the reasons underlying these improvements is the chronic effects of PAP, which explains the increased performance in explosive movements following resistance training.

CT induces adaptations both in neural activation patterns and muscle capacity, enhancing motor unit firing rates and muscle power output (17). Such neuromechanical improvements are directly transferable to basketball-specific movements, thereby supporting the observed gains in CoD speed and vertical jump performance (15). Another study supporting this situation is a meta-analysis conducted by Pagaduan and Pojskić (32). In the analysis in question, it was reported that CT significantly improved vertical jump performance, which was superior to the gains achieved with plyometric training alone. Similarly, Trzaskoma et al. (65) reported that adding weight training components to a training program resulted in higher jumping performance than traditional plyometric training. The mechanisms underlying these improvements include improved neural activation patterns during the stretch-shortening cycle and increased muscle contraction capacity (66). In addition, Cormie et al. (66) stated that adaptations occurring in the eccentric phase of the jumping movement are critical for the effectiveness of plyometric performance and contribute significantly to the improvement in jumping abilities after CT. These findings indicate that successful training interventions change both neural activation and muscle capacity; the firing rates of motor units increase, and the power production capacity of the muscle improves. This is also consistent with the findings of the meta-analysis conducted by Ramírez-Campillo et al. (14), which reported that various plyometric jump training sessions combined with strength training led to significant improvements in vertical jump performance in athletes.

## 6 Practical applications

The results of this systematic review and meta-analysis indicate that CT, which integrates strength and plyometric exercises, significantly enhances CoD speed and vertical jump performance in basketball players. Coaches and practitioners can strategically implement CT across different phases of the season, employing higher-volume sessions during the pre-season to build foundational strength and power, moderate-volume, high-intensity sessions in-season to maintain performance, and progressive overload during the off-season to maximize adaptations. It is recommended that resistance exercises precede plyometric drills to utilize post-activation potentiation, thereby promoting rapid force application during basketball-specific movements such as jump shots, rebounds, and defensive slides. As the first meta-analysis focused exclusively on CT in basketball, this study provides an evidence-based framework for designing targeted, sport-specific training programs. Furthermore, these findings can inform the development of standardized CT protocols, guide future intervention designs, and highlight research gaps, particularly concerning long-term effects, comparisons with other training modalities, and implications for injury prevention. Overall, this review offers actionable insights for coaches and practitioners seeking to optimize performance through safe, scientifically grounded, and effective training interventions.

## 7 Strengths and limitations

This study offers several strengths, including its focus as one of the first systematic reviews and meta-analyses specifically examining CT in basketball players, the inclusion of only RCTs to ensure methodological rigor, and a comprehensive search strategy across multiple databases. These factors enhance the reliability, transparency, and practical relevance of the findings for coaches and practitioners. Nevertheless, several limitations should be acknowledged. The small number of included studies (seven RCTs) limits the generalizability of the findings. Considerable variability existed in CT protocols, including differences in exercise selection, intensity, frequency, volume, duration, and recovery periods, which may introduce methodological heterogeneity. Participant characteristics, such as age, sex, and training experience, also varied across studies, and some studies lacked detailed reporting on training intensity, progression, or adherence, restricting deeper subgroup or moderator analyses. Additionally, the review was limited to studies published in English, and potential publication bias cannot be entirely excluded. Finally, none of the included studies provided long-term follow-up data on performance maintenance or injury risk.

To advance this line of research, future studies should aim to include larger and more diverse samples of basketball players across different competitive levels, ages, and genders. Standardizing CT intervention protocols in terms of exercise selection, intensity, and progression would allow for more precise comparisons and stronger meta-analytic conclusions. Greater transparency and consistency in reporting methodological details are also needed to facilitate replication and moderator analyses. Additionally, longitudinal studies examining the long-term effects of CT on both performance and injury prevention could provide more comprehensive insights. Finally, integrating physiological and biomechanical measures alongside performance outcomes may help clarify the mechanisms underlying CT adaptations and enhance the translational value for coaches and practitioners.

## 8 Conclusion

This meta-analysis confirms that CT, which integrates strength and plyometric exercises, significantly enhances key physical performance attributes in basketball players namely, jump performance and CoD speed. The within-group analysis showed marked improvements in both domains, indicating that CT effectively targets the explosive power and agility essential for basketball performance. Additionally, between-group comparisons with active controls demonstrated the superiority of CT, further validating its effectiveness over traditional training approaches. These findings hold practical significance for coaches and practitioners seeking to optimize sport-specific athletic performance. Enhancements in CoD speed and jumping ability are critical for executing rapid transitions, directional changes, and explosive movements during gameplay. CT appears to stimulate neuromuscular adaptations that translate well into these sport-specific skills, thereby supporting its integration into basketball conditioning programs. Moreover, the consistency of findings across multiple RCTs adds robustness to the conclusion that CT is an effective method for improving physical attributes central to basketball. Coaches are encouraged to implement CT protocols as part of their athletes' training regimens to maximize on-court performance. Future research should aim to explore long-term effects, gender differences, and positional demands to further refine CT's application in basketball and broader athletic populations.

## Data Availability

The original contributions presented in the study are included in the article/Supplementary Material, further inquiries can be directed to the corresponding authors.
